# The Role of Genetic Risk Score in Predicting the Risk of Hypertension in the Korean population: Korean Genome and Epidemiology Study

**DOI:** 10.1371/journal.pone.0131603

**Published:** 2015-06-25

**Authors:** Nam-Kyoo Lim, Ji-Young Lee, Jong-Young Lee, Hyun-Young Park, Myeong-Chan Cho

**Affiliations:** 1 Division of Cardiovascular and Rare Diseases, Korea National Institute of Health, Cheongju, Republic of Korea; 2 Division of Structural and Functional Genomics, Korea National Institute of Health, Cheongju, Republic of Korea; 3 Cardiovascular Research Institute and Cardiovascular Genome Center, Yonsei University Health System, Seoul, Republic of Korea; 4 Department of Cardiology, College of Medicine, Chungbuk National University, Cheongju, Republic of Korea; McMaster University, CANADA

## Abstract

Hypertension is regarded as a multifactorial disease with a modest contribution of genetic factors and strongly affected by environmental factors. Recent genome-wide association studies have identified specific loci associated with high blood pressure (BP) and hypertension. This study aimed to examine the association between the genetic risk score (GRS), a linear function of multiple single nucleotide polymorphisms (SNPs) associated with hypertension, and high BP and prevalent hypertension at baseline examination and to evaluate the efficacy of the GRS for predicting incident hypertension with longitudinal data in Korean subjects. Data for 8,556 participants, aged 40 to 69, in a community-based cohort study were analyzed. Unweighted GRS (cGRS) and weighted GRS (wGRS) were constructed from 4 SNPs related to high BP or hypertension in previous genome-wide association and its replication studies for the Korean middle-aged population. Cross-sectional analysis (n=8,556) revealed that cGRS was significantly associated with prevalent hypertension (odds ratio=1.15 per risk allele; 95%CI, 1.09-1.20). Additionally, the odds ratios (ORs) of prevalent hypertension for those who in medium and the highest tertile compared with those who in the lowest tertile of wGRS were 1.31 (95% CI, 1.15-1.50) and 1.59 (95%CI, 1.38-1.82), respectively. In a longitudinal analysis (n=5,632), participants in the highest tertile of wGRS had a 1.22-fold (OR=1.22, 95%CI, 1.02‒1.46) greater risk of incident hypertension relative to those in the lowest tertile, after adjusting for a number of confounding factors. However, wGRS topped with traditional risk factors had no significant effect on discrimination ability (c-statistics with and without wGRS were 0.811 and 0.810, P=0.1057). But, reclassification analysis showed that the addition of GRS to the model with conventional risk factors led to about 9% significant increment in category-free net reclassification improvement. GRSs based on 4 SNPs were independently associated with hypertension and may provide a statistically significant improvement over the existing model for prediction of incident hypertension.

## Introduction

In recent years, large international consortia have used genome-wide association studies (GWASs) to identify several genetic susceptibility variants for hypertension and/or elevated blood pressure (BP) in different ethnic populations [1–5]. To date, although more than 40 hypertension-related loci have been identified, the ability to discriminate genetically between individuals with high BP and normal BP remains limited. In the Korean population, the most strong association involved chromosome 12q21 and variants near the ATP2B1 gene for systolic blood pressure [6] and several variants related with hypertension were replicated in other independent Koreans [7]. This novel variants were located in the 50-region upstream of the plasma membrane calcium transporting ATPase1 (ATP2B1) gene. However, because of a relatively low effect size of single SNP, combining the effects of genetic risk variants into genetic risk scores (GRSs) may improve in risk prediction.

An increase in the genetic risk score (GRS), which is derived from single-nucleotide polymorphisms (SNPs) identified by GWASs, is associated with the incidence of coronary events, stroke, and hypertension in the Caucasian population [8]. Recent studies have demonstrated significant improvements in coronary risk prediction using GRS for coronary artery disease and hypertension [9–11]. In addition, several research groups have attempted to consolidate genetic risk information and traditional risk variables with the aim of improving disease classification [12, 13].

However, few of these studies identified a potential use for GRS [14–16]. Recent GWASs conducted in Asia focused primarily on cross-sectional analysis; therefore, they could not predict the incidence of hypertension or BP change over time [7, 17–20]. The present study used data from middle-aged Korean males and females to determine whether GRS is independently associated with prevalent hypertension and high BP, and whether GRS can be used to predict the new onset of hypertension within a ~4-year follow-up period.

## Methods

### Subjects and genotyping

**Fig 1:**
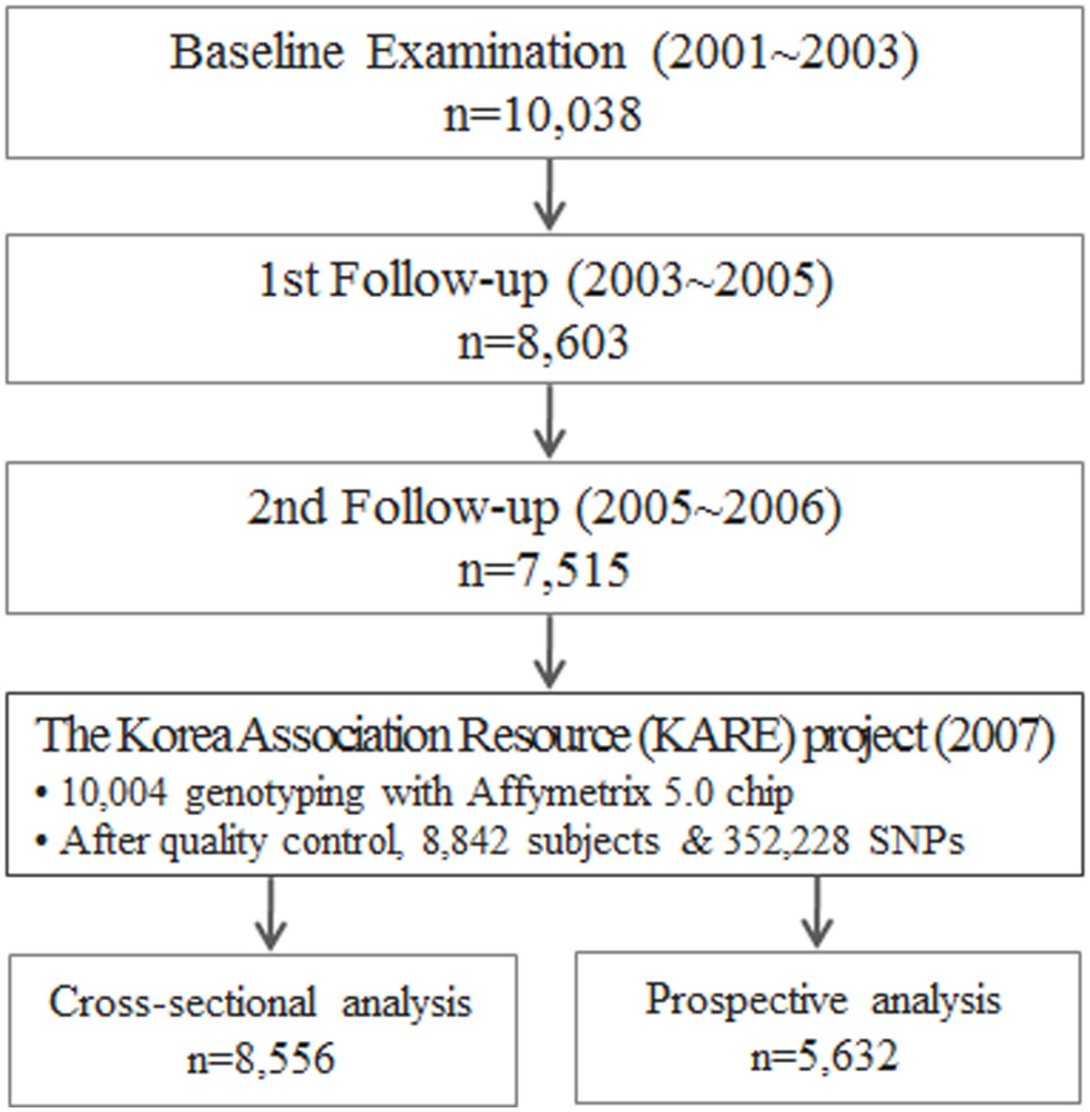
Study flow chart and analysis sets from the KoGES and KARE projects.

The Korean Genome and Epidemiology Study (KoGES) is an ongoing community-based cohort study that started in 2001 with the support of the Korean National Institute of Health. The study has been described in detail previously [21]. Briefly, 10,038 participants (aged 40–69 years) were enrolled. A baseline examination was performed between 2001 and 2003, and follow-up examinations were conducted at 2-year intervals. Participants resided in the Ansan and Ansung regions of Korea. The current study used baseline and first and second follow-up examination data (Fig 1).

The Korea Association Resource (KARE) project was established in 2007 to perform a large-scale GWAS analysis of the aforementioned Ansung and Ansan cohorts. In total, 10,004 KARE study participants were genotyped using the Affymetrix Genome-Wide Human SNP array 5.0, and individual genotypes were identified by applying the Bayesian Robust Linear Modeling using the Mahalanobis distance genotyping algorithm. After quality control, data from 8,842 subjects and 352,228 SNPs were used for analysis. The KARE study has been described in detail previously [6].

For cross-sectional analysis of prevalent hypertension and elevated BP, 8,556 participants were analyzed at baseline. For prospective analysis, data were obtained at baseline as well as at the 2- and 4-year follow-up examinations. At this point, 1,983 participants with prevalent hypertension at baseline (including those taking antihypertensive medication and those with a systolic blood pressure (SBP) ≥140 mm Hg or a diastolic blood pressure (DBP) ≥90 mm Hg) and 147 participants with prevalent cardiovascular disease or a serum creatinine level of >2 mg/dL, were excluded. 794 participants for whom no complete data were available were also excluded. Therefore, 5,632 participants were eligible for prospective analysis. The study protocol was approved by the Institutional Review Board of the Korea Centers for Disease Control and Prevention (KCDC). All participants signed written informed consent form approved by the Human Subjects Committee before participating in this study.

### Measurements and surveys

Anthropometric measurements were obtained for each participant, and blood samples were drawn for biochemical analysis. A questionnaire was used to collect demographic information, lifestyle information (including smoking status and alcohol consumption), as well as personal and family medical histories. “Current smoking” was defined as smoking at least one cigarette per day for at least the previous year. The participants were also classified as non-, ex-, or current drinkers. Body mass index (BMI) was calculated by dividing body weight (kg) by height squared (m^2^). BP was taken by trained technicians using mercury sphygmomanometers (Baumanometer-Standby; W.A. Baum Co. Inc., Copiague, NY, USA). SBP and DBP were defined as the average of three times readings obtained while supine position after a minimum 5-min rest. Participants were classified as hypertensive if they had a SBP ≥140 mmHg or a DBP ≥90 mmHg or were taking antihypertensive medication at the time of the baseline examination.

### Construction of GRSs

Two types of GRS were constructed based on 4 SNPs that were reported in the study using recent GWAS and the replication study using the KARE project and Health 2 cohort data [6, 7]. First, the unweighted GRS (cGRS) was determined by a simple summation of the number of risk alleles from the 4 SNPs. Second, a weighted GRS (wGRS) was created by multiplying each allele number by the beta coefficients suggested by the multiple regression model used in a previous study [7]. The detailed method used to construct the GRS is presented in the supporting information (S1 File).

### Statistical analysis

Multiple linear regression and logistic regression analyses were used for cross-sectional and prospective analysis of whether GRS is an independent risk factor for hypertension and high BP. We chose the covariates among the traditional risk factors that were well known as risk factors of cardiovascular diseases and could be simply obtained by the clinical test and questionnaire of our studies. Odds ratios (ORs) for prevalence and incidence of hypertension according to cGRS, wGRS, and tertile of wGRS were obtained after adjustment for traditional risk factors. Model 1 was adjusted for age and sex. Model 2 was adjusted model 1 plus other cardiovascular risk factors, including smoking status, SBP, parental history of hypertension, and body mass index (BMI).

The accuracy of the risk models was compared in terms of calibration, discrimination, and reclassification. To compare the discrimination ability of the models, we calculated the area under the receiver operating characteristic curves (AROC), known as the c-statistic, which assesses the ability of the model to distinguish those who experience hypertension from those who do not. A goodness-of-fit test based on the Hosmer–Lemeshow χ^2^-statistic was used to assess calibration; it measures how closely the predicted probability agrees with the actual observed probability [22]. The user-category and category-free net reclassification improvement (NRI) and integrated discrimination improvement (IDI) were calculated to evaluate improvements in the reclassification for both risk models with and without the addition of GRS [23, 24].

Finally, the internal validity of the model was assessed by examining performance using the k-fold cross validation technique [24, 25] and the *a posteriori* power. A two-tailed P-value less than 0.05 was deemed to indicate statistical significance. Statistical analyses were performed using the SAS software (version 9.3; SAS Institute, Cary, NC, USA).

## Results

The results of the goodness-of-fit test for Hardy-Weinberg equilibrium for the 4 SNPs used to calculate the GRS are summarized in Table 1. Among 8,556 participants (aged 40 to 69 years at baseline) at baseline, the prevalence of hypertension was 23.2% (22.3% in men and 23.9% in women). Several baseline characteristics of data for cross-sectional and prospective analyses are shown in Table 2.

**Table 1:** Hardy-Weinberg equilibrium and genotype frequencies for 4 SNPs included in the genetic risk score.

Chr. No.						Genotype Frequency			
	Gene name	SNP ID	Minor Allele	Major Allele	MAF	0	1	2	P-value†
8	CSMD1	rs995322	T	C	0.360	3543	3874	1139	0.1198
12	ATP2B1	rs17249754	A	G	0.373	3391	3939	1226	0.1327
15	CSK	rs1378942	A	C	0.172	5852	2460	244	0.4513
17	ARSG	rs12945290	G	A	0.133	6452	1931	173	0.0439

Abbreviation: Chr, chromosome; SNP, single nucleotide polymorphism; MAF, minor allele frequency;

^†^P-value for Hardy-Weinberg Equilibrium.

**Table 2:** Baseline characteristics of the study participants for cross-sectional and prospective analysis.

Variable	Data for cross-sectional analysis	Data for prospective analysis
N	8,556	5,632
Age, years	52.2±8.9	50.9±8.6
Male gender, %	47.8	48.5
SBP, mm Hg	117.6±18.3	111.0±12.6
DBP, mm Hg	75.1±11.6	71.6±9.1
Currently smoking, %	25.5	26.3
Body mass index, kg/m^2^	24.6±3.1	24.3±3.0
Diabetes mellitus, %	14.0	10.9
Alcohol intake, %		
None	46.3	45.5
Former drinker	6.4	6.1
Current drinker	47.3	48.5
Parental history of hypertension, %	16.6	14.8
Hypertension at baseline, %	23.2	-

Abbreviations: SBP, systolic blood pressure; DBP, diastolic blood pressure; GRS, genetic risk score.

The results of multiple logistic and linear regression models used for cross-sectional analysis are shown in Tables 3 and 4. Table 3 shows that, after adjusting for traditional risk factors, both cGRS and wGRS were significantly associated with prevalent hypertension, with ORs of 1.15 (95% CI, 1.09–1.20) and 1.12 (95% CI, 1.08–1.16), respectively. In addition, individuals in the highest wGRS tertile were 59% more likely to be hypertensive than those in the lowest tertile (OR = 1.59; 95% CI, 1.38–1.82; P<0.0001).

**Table 3:** Association between GRSs and hypertension for cross-sectional analysis.

Genetic Risk Score	Multiple logistic regression analysis			
	Model 1		Model 2	
	OR (95% CI)	P-value	OR (95% CI)	P-value
cGRS	1.14 (1.09–1.19)	<0.0001	1.15 (1.09–1.20)	<0.0001
wGRS	1.12 (1.08–1.16)	<0.0001	1.12 (1.08–1.16)	<0.0001
Tertile of wGRS				
Medium vs Low	1.32 (1.16–1.50)	<0.0001	1.31 (1.15–1.50)	<0.0001
High vs Low	1.59 (1.39–1.81)	<0.0001	1.59 (1.38–1.82)	<0.0001

Abbreviations: CI, confidence interval; OR, odds ratio.

Model 1 was adjusted by age and sex; and Model 2 was adjusted by age, sex, current status of smoking, parental history of hypertension, and body mass index.

**Table 4:** Association between GRS and DBP and SBP at baseline.

Variable		Multiple Logistic and Linear Regression Analysis			
		Model 1		Model 2	
	Type of GRS	Beta (SEM)	P-value	Beta (SEM)	P-value
SBP^§^	cGRS	1.38 (0.16)	<0.0001	1.32 (0.16)	<0.0001
	wGRS	1.05 (0.12)	<0.0001	1.01 (0.12)	<0.0001
	Tertile of wGRS				
	Low vs. Medium	2.54 (0.50)	<0.0001	2.02 (0.45)	<0.0001
	Low vs. High	3.54 (0.47)	<0.0001	3.11 (0.42)	<0.0001
DBP^§^	cGRS	0.70 (0.07)	<0.0001	0.65 (0.06)	<0.0001
	wGRS	0.99 (0.12)	<0.0001	0.94 (0.12)	<0.0001
	Tertile of wGRS				
	Low vs. Medium	1.25 (0.34)	0.0002	1.15 (0.33)	0.0004
	Low vs. High	2.04 (0.31)	<0.0001	1.92 (0.30)	<0.0001

Abbreviations: GRS, genetic risk score; SBP, systolic Blood Pressure; DBP, diastolic Blood Pressure; SEM, standard error of mean; beta, parameter estimate of multiple linear regression analysis. Model 1 was adjusted by age and gender; Model 2 was adjusted by age, gender, current status of smoking, parental history of hypertension, and body mass index.

^§^SBP+15 and DBP+10, if subject was treated with antihypertensive medication.

Both types of GRSs were strongly associated with high SBP and DBP, even after adjustment (Table 4). SBP was significantly higher in individuals in the middle (β = 2.02, P<0.0001) and the highest (β = 3.11, *P*<0.0001) wGRS tertiles than those in the lowest tertile. Similar associations between GRSs and DBP were also observed (β = 0.65, P<0.0001 for cGRS; β = 0.94, P<0.0001 for wGRS).

**Fig 2:**
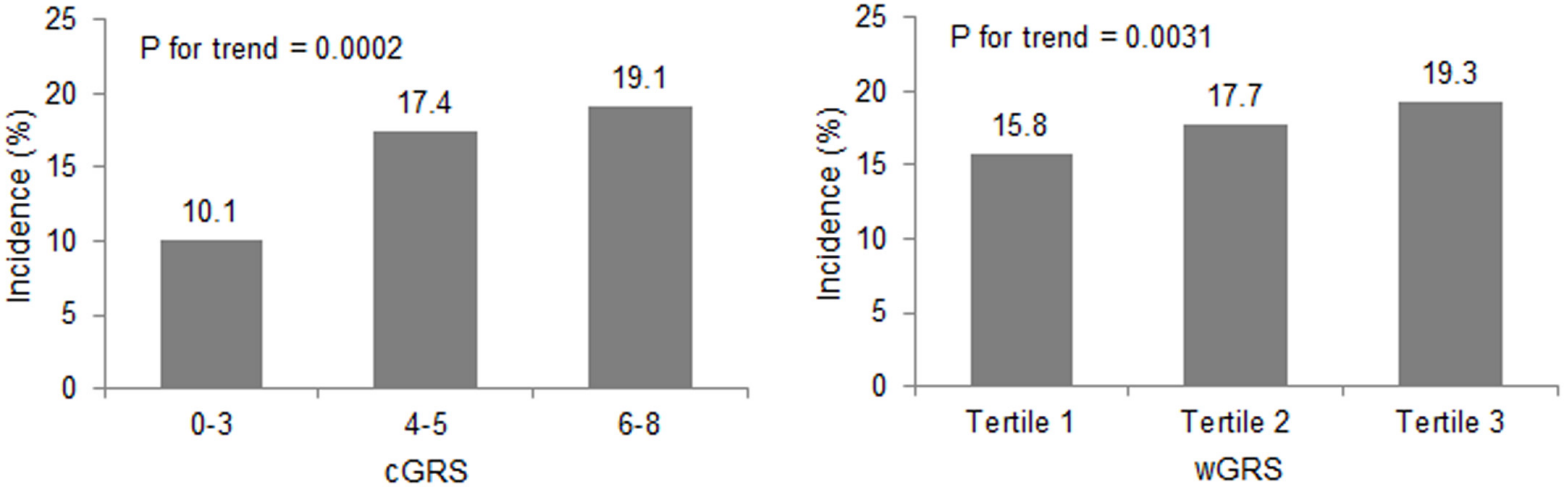
Cumulative incidence of hypertension according to cGRS and wGRS. Abbreviations: cGRS, unweighted genetic risk score; wGRS, weighted genetic risk score. P for trend means p-value for Cochran-Armitage linear trend test.

The prospective analyses included data obtained from 5,632 participants at baseline and at the 2- or 4-year follow-up examination (Fig 1). Table 5 presents the association between incidence of hypertension and GRSs. After adjusting for common risk factors, an increase in cGRS and wGRS led to an increase in the risk of hypertension incidence (OR = 1.11; 95% CI, 1.04–1.19 and OR = 1.09; 95% CI, 1.04–1.15, respectively). In addition, between subjects in the highest tertile of the wGRS, the OR for incident hypertension with respect to the lowest tertile was 22% higher (OR = 1.22; 95% CI, 1.02–1.46). The association between other traditional risk factors and hypertension incidence is presented in S1 Table. Cumulative incidence of hypertension increased linearly from 10.1% for cGRS≤3 to 19.1% for cGRS≥6. A similar pattern was also observed according to the tertile of wGRS (Fig 2).

**Table 5:** Multiple adjusted hazard ratios for hypertension incidence after 4-year follow-up.

Genetic Risk Score	Model 1		Model 2	
	OR (95% CI)	*P-*value	OR (95% CI)	*P*-value
cGRS	1.14 (1.08–1.21)	<0.0001	1.11 (1.04–1.19)	0.0010
wGRS	1.12 (1.06–1.17)	<0.0001	1.09 (1.04–1.15)	0.0010
Tertile of wGRS				
Medium vs. Low	1.21 (1.00–1.45)	0.0456	1.18 (0.96–1.44)	0.1104
High vs. Low	1.29 (1.09–1.52)	0.0028	1.22 (1.02–1.46)	0.0306

Abbreviations: OR, odd ratio; CI, confidence Interval; wGRS, weighted genetic risk score; cGRS, count genetic risk score. Model 1 was adjusted by age and gender at baseline; Model 2 was adjusted by age, gender, systolic blood pressure, current status of smoking, parental history of hypertension, and body mass index.

We tested the calibration, discrimination, and reclassification of the constructed model using the Hosmer–Lemeshow goodness-of-fit test, c-statistics, and NRI and IDI, respectively (Table 6). The calibration in the models with and without GRS produced favorable results (Table 6 and S1 Fig). The c-statistic was 0.810 (95% CI, 0.796–0.824) for the model without wGRS and 0.811 (95% CI, 0.797–0.825) for the model with wGRS, indicating similar and good discrimination ability for both (Table 6). To examine whether the reclassification ability was improved by adding wGRS to the model, we examined the NRI using both category-free and pre-specified category parameters (<4%, 4% to <8%, 8% to <12%, 12% to <16%, 16% and higher). We found that wGRS contributed an 8.9% (95% CI, 2.0%-15.7%, P = 0.0113) improvement in category-free NRI and about 2.0% (95% CI, 0.0%-3.7%, P = 0.0495) in user-category NRI. The IDI also yielded significant improvement in reclassification (Table 6). Internal validation using the fivefold cross-validation technique was performed to test the performance of the models when used for new cases of hypertension. The mean value for the c-statistic was 0.811 (ranged from 0.809 to 0.816), which represented high discriminatory performance in the split subsets. Based on the sample size of multiple logistic regression on longitudinal data and its results, we were able to detect OR of at least 1.2 with the power 90%.

**Table 6:** Reclassification ability with include weighted GRS to the risk prediction model.

Measurements	Risk Prediction Model*		
	Without wGRS	With wGRS	P-value
**Calibration, χ^2^** **†**	6.919	5.711	NS‡
**AROC (95% CI)**	0.810 (0.796–0.824)	0.811 (0.797–0.825)	0.1057
**Category-free NRI (95% CI)**	0.089 (0.020, 0.157)		0.0113
**User category NRI (95% CI)** **††**	0.019 (0.000, 0.037)		0.0495
**IDI (95% CI)**	0.002 (0.000, 0.004)		0.0131

Abbreviations: GRS, genetic risk score; CI, confidence interval; NRI, net reclassification improvement; IDI, integrated discrimination improvement.

*Model adjusted by age, gender, parental history of hypertension, smoking status, SBP, DBP, and interaction of age by DBP.

^†^Hosmer and Lameshow’s goodness-of-fit test.

^††^User category of risk was defined as follows: <4%, 4% to <8%, 8% to <12%, 12% to <16%, ≥16%.

^‡^Neither model was significant according to the goodness-of-fit test.

## Discussion

Whether the aggregation into a single metric of several genetic variants can help to explain incidence of hypertension or cardiovascular diseases has been controversial. The present study examined the association between GRS, derived from 4 SNPs related to high BP and prevalent hypertension identified in a previous large GWAS, and the incidence of hypertension among individuals aged 40–69 years. We also determined whether the ability of models to predict the risk of incident hypertension was improved by the addition of GRS. The models were constructed using data for six commonly used traditional risk variables (age, gender, SBP, current smoking status, family history of hypertension, BMI) and one genetic variable (cGRS or wGRS derived from the 4 SNPs). Excepting GRS, these variables can be easily evaluated and calculated using a simple questionnaire and non-invasive medical check.

We found that, independent of whether cGRS or wGRS was used, individuals with a high GRS have higher SBPs and DBPs and a higher risk of prevalent hypertension than those with a low GRS at baseline. Each risk allele contributed to an increase in OR for an incident hypertension of about 1.11 times at a 4-year follow-up. The incidence of hypertension displayed increasing patterns, according to the category of cGRS and tertile of wGRS (Fig 2).

Additionally, GRS was independently associated with high BP and hypertension incidence after adjusting for common risk variables, including a family history of hypertension (Table 5 and S1 Table). The degree of association of GRS with hypertension incidence was similar to that with current smoking status but was weaker than the association with a family history of hypertension. Despite these statistically significant associations, the addition of GRS (alongside traditional risk variables) to the common model did not improve discrimination and reclassification in terms of the incidence of hypertension.

Several studies have examined the effect of GRS consisting of candidate SNPs, which were related with high BP and/or cardiovascular disease (CVD) revealed by large GWASs [14, 16, 26]. A recent longitudinal study of Swedish individuals conducted by Fava *et al*. suggested that GRS was positively associated with elevated BP and the incidence of hypertension, independent of traditional risk variables; however, the results of discrimination analysis showed that the prediction model with GRS was no more effective than the conventional model without GRS [14]. Another study, using data obtained from a population-based Finnish cohort as they grew from children to adults, indicated that GRS derived from 13 SNPs is associated with cardiovascular events, but improvement in risk prediction was achieved by addition of GRS to traditional risk factors of CVD [26]. The results of our study imply a conservative use of GRS topped with traditional risk factors, accordant with the insistence on limited clinical use of GRS by Fava *et al*. To date, most studies have shown that GRS improves discrimination with respect to traditional risk variables as assessed by ROC curves, although a few studies have suggested that GRS improves risk reclassification, albeit minimally. The traditional risk factors including the anthropometric and environmental factors play more important role than genetic information (S1 Table). But, the result of our study shows that the addition of GRS to traditional risk factors may help restrictively improve ability in terms of the reclassification.

The strengths of the present study include the use of large community-based cohorts, derived from both urban and rural areas in Korea, and the inclusion of appropriate follow-up examinations. However, several limitations exist. First, the SNPs were used without considering their possible interactions with other genetic variants or other environmental factors that could explain physiological BP homeostasis. Although the included SNPs were validated in Korean individuals, other SNPs from candidate genes or biological pathway-based approaches could be used in a more suitable GRS. Second, the results from our cohort cannot be generalized to individuals with different genetic backgrounds and other epidemiological and environmental settings. Future studies are required to determine whether different scores are needed for individuals of different ethnicity, or whether other risk factors should be considered before applying the GRS. Third, internal validation using a bootstrap method is of limited value when used to determine the generalizability of the model [27]. Therefore, external validation is needed before the prediction model can be used clinically.

## Conclusion

The suitability of GRS for predicting the risk of incident hypertension has been examined in several ethnic groups, particularly those of European descent. However, few studies of Asian populations have been conducted. In the current Korean cohort study, we found that GRSs derived from 4 SNPs were independently associated with an increased BP or hypertension and were highly associated with an increased risk of incident hypertension, even after adjusting for traditional risk factors. While adding GRS on the traditional risk factors did not result in improvement of discrimination ability, the reclassification analysis revealed that an addition of GRS produces a statistical significant effect.

## Supporting Information

S1 Fig Calibration graph and Hosmer and Lameshow's goodness-of-fit test for the models with and without GRSs. (TIF)

S1 File Calculation of genetic risk scores (DOCX)

S1 Table Multiple logistic regression model and odds ratios for incidence of hypertension. (DOCX)
