# Supplementary figures and images for: The Role of Genetic Risk Score in Predicting the Risk of Hypertension in the Korean population: Korean Genome and Epidemiology Study
Source: PLoS One. 2015 Jun 25;10(6):e0131603. doi: 10.1371/journal.pone.0131603 (PMC4482533; doi:10.1371/journal.pone.0131603)

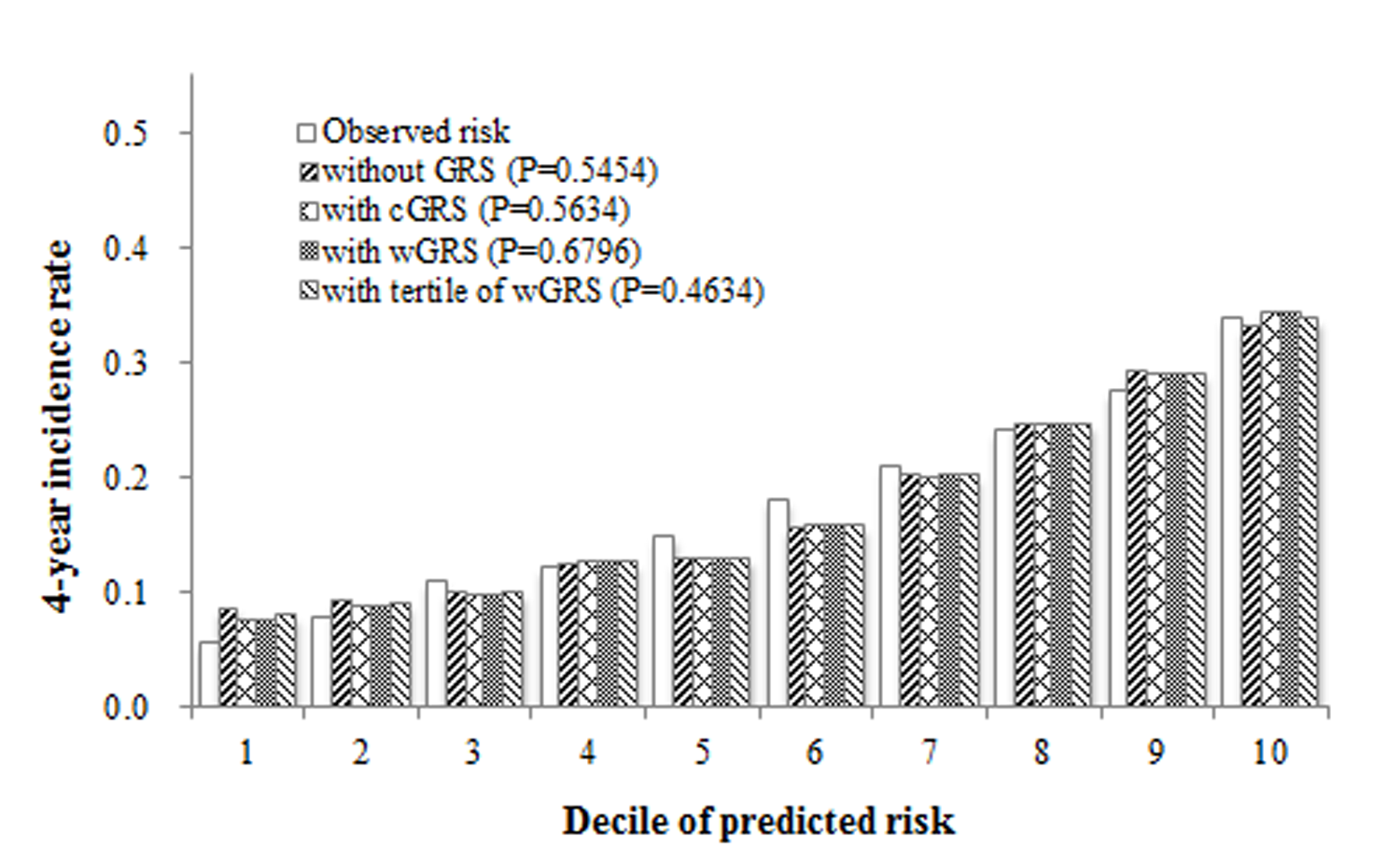

Supplement: S1 Fig — (TIF) [file pone.0131603.s001.tif]
