# Supplementary material for: The Role of Genetic Risk Score in Predicting the Risk of Hypertension in the Korean population: Korean Genome and Epidemiology Study
Source: PLoS One. 2015 Jun 25;10(6):e0131603. doi: 10.1371/journal.pone.0131603 (PMC4482533; doi:10.1371/journal.pone.0131603)
Supplement: S1 File — (DOCX) [file pone.0131603.s002.docx]

**S1 File. Calculation of genetic risk scores**

In order to create the unweighted GRS (cGRS) and weighted GRS (wGRS), we used the following linear functions using 4 SNPs:

**1) cGRS**

cGRS= rs995322 +rs17249754+ rs1378942+rs12945290 (Each SNP has value from 0 to 2 according to the number of risk allele)

**2) wGRS for hypertension**

wGRS=w1*rs995322+w2*rs17249754+w3*rs1378942+w4*rs12945290, where w1=1.13, w2=1.27, w3=1.36, and w4=1.18.

**3) wGRS for systolic blood pressure**

wGRS=ws1*rs995322+ ws2*rs17249754 +ws3*rs1378942+ws4*rs12945290, where ws1=1.14, ws2=1.34, ws3=1.42, and ws4=1.53.

**4) wGRS for diastolic blood pressure**

wGRS=wd1*rs995322+wd2*rs17249754+wd3*rs1378942+wd4*rs12945290, where wd1=0.76, wd2=0.90, wd3=0.93, and wd4=0.90.
