# Supplementary material for: The Role of Genetic Risk Score in Predicting the Risk of Hypertension in the Korean population: Korean Genome and Epidemiology Study
Source: PLoS One. 2015 Jun 25;10(6):e0131603. doi: 10.1371/journal.pone.0131603 (PMC4482533; doi:10.1371/journal.pone.0131603)
Supplement: S1 Table — (DOCX) [file pone.0131603.s003.docx]

**S1 Table.** Multiple logistic regression model and odds ratios for incidence of hypertension.

| **variables** | **Model 1** | |  | **Model 2** | |  | **Model 3** | |  | **Model 4** | |
| --- | --- | --- | --- | --- | --- | --- | --- | --- | --- | --- | --- |
|  | **OR (95% CI)** | **P-value** |  | **OR (95% CI)** | **P-value** |  | **OR (95% CI)** | **P-value** |  | **OR (95% CI)** | **P-value** |
| **Age, y** | 1.04 (1.03-1.05) | <0.0001 |  | 1.04 (1.03-1.05) | <0.0001 |  | 1.04 (1.03-1.05) | <0.0001 |  | 1.04 (1.03-1.05) | <0.0001 |
| **Sex, Women** | 0.95 (0.80-1.14) | 0.6008 |  | 0.95 (0.80-1.14) | 0.6074 |  | 0.95 (0.80-1.14) | 0.6086 |  | 0.96 (0.80-1.15) | 0.6238 |
| **SBP** | 1.10 (1.09-1.11) | <0.0001 |  | 1.10 (1.09-1.10) | <0.0001 |  | 1.10 (1.09-1.10) | <0.0001 |  | 1.10 (1.09-1.10) | <0.0001 |
| **Smoking status** | 1.24 (1.01-1.52) | 0.0381 |  | 1.24 (1.01-1.52) | 0.0368 |  | 1.24 (1.01-1.52) | 0.0364 |  | 1.24 (1.01-1.52) | 0.0369 |
| **Parental history** | 1.47 (1.19-1.82) | 0.0004 |  | 1.47 (1.19-1.82) | 0.0004 |  | 1.47 (1.19-1.82) | 0.0004 |  | 1.48 (1.19-1.83) | 0.0003 |
| **BMI** | 1.09 (1.06-1.12) | <0.0001 |  | 1.09 (1.07-1.12) | <0.0001 |  | 1.09 (1.07-1.12) | <0.0001 |  | 1.09 (1.07-1.12) | <0.0001 |
| **cGRS** | - |  |  | 1.11 (1.04-1.19) | 0.0010 |  | - |  |  | - |  |
| **wGRS** | - |  |  | - |  |  | 1.09 (1.04-1.15) | 0.0010 |  | - |  |
| **wGRS** | - |  |  | - |  |  | - |  |  | - |  |
| **Tertile 1** | - |  |  | - |  |  | - |  |  | 1(Ref.) |  |
| **Tertile 2** | - |  |  | - |  |  | - |  |  | 1.18 (0.96-1.44) | 0.1104 |
| **Tertile 3** | - |  |  | - |  |  | - |  |  | 1.22 (1.02-1.46) | 0.0306 |
| **c-statistics** | 0.810 | |  | 0.811 | |  | 0.811 | |  | 0.811 | |
| **Hosmer-Lameshow’s χ^2^** | 6.919 | |  | 6.754 | |  | 5.711 | |  | 7.699 | |

Abbreviations: SBP, systolic blood pressure; DBP, diastolic blood pressure; BMI, body mass index; OR, odds ratio; NI, not included in models; CI, confidence interval for odds ratio; Tertile 1,2,3= bottom, middle, and top tertile, respectively.
